# Assessment of the Carbon Monoxide Metabolism of the Hyperthermophilic Sulfate-Reducing Archaeon *Archaeoglobus fulgidus* VC-16 by Comparative Transcriptome Analyses

**DOI:** 10.1155/2015/235384

**Published:** 2015-08-06

**Authors:** William P. Hocking, Irene Roalkvam, Carina Magnussen, Runar Stokke, Ida H. Steen

**Affiliations:** Department of Biology, Centre for Geobiology, University of Bergen, 5020 Bergen, Norway

## Abstract

The hyperthermophilic, sulfate-reducing archaeon, *Archaeoglobus fulgidus*, utilizes CO as an energy source and it is resistant to the toxic effects of high CO concentrations. Herein, transcription profiles were obtained from *A. fulgidus* during growth with CO and sulfate or thiosulfate, or without an electron acceptor. This provided a basis for a model of the CO metabolism of *A. fulgidus*. The model suggests proton translocation by “Mitchell-type” loops facilitated by Fqo catalyzing a Fd_red_:menaquinone oxidoreductase reaction, as the major mode of energy conservation, rather than formate or H_2_ cycling during respiratory growth. The bifunctional CODH (*cdhAB-2*) is predicted to play an ubiquitous role in the metabolism of CO, and a novel nitrate reductase-associated respiratory complex was induced specifically in the presence of sulfate. A potential role of this complex in relation to Fd_red_ and APS reduction is discussed. Multiple membrane-bound heterodisulfide reductase (DsrMK) could promote both energy-conserving and non-energy-conserving menaquinol oxidation. Finally, the FqoF subunit may catalyze a Fd_red_:F_420_ oxidoreductase reaction. In the absence of electron acceptor, downregulation of F_420_H_2_ dependent steps of the acetyl-CoA pathway is linked to transient formate generation. Overall, carboxidotrophic growth seems as an intrinsic capacity of *A. fulgidus* with little need for novel resistance or respiratory complexes.

## 1. Introduction

Carboxidotrophs grow chemolitoautotrophically on carbon monoxide (CO) and are considered to hold a vital niche in terrestrial and marine thermophilic ecosystems [1]. CO-utilizing microorganisms include aerobic bacteria, phototrophic purple nonsulfur bacteria, acetogens, methanogens, and hydrogenogenic bacteria and archaea, as well as sulfate-reducing prokaryotes (SRP) [1, 2]. The hyperthermophile,* Archaeoglobus fulgidus*, is so far the only known carboxydotrophic sulfate-reducing archaeon and has a high tolerance for CO, growing at more than 200 kPa of CO [2, 3]. Furthermore,* A. fulgidus* grows as an acetogen with CO in the absence of an external electron acceptor [2]. Beside utilizing CO,* A. fulgidus* grows with H_2_ and formate and a wide variety of simple and complex organic compounds [4–7].

Growth on CO requires the enzyme carbon monoxide dehydrogenase (CODH) that catalyzes the reversible conversion between CO and CO_2_. Multiple CODHs are often present in carboxidotrophs potentially facilitating separate, CO oxidation and CO_2_-assimilation reactions [8]. Genomic sequencing of* A. fulgidus* suggests that three [Ni-Fe]-CODHs are present, a “bacterial” monomeric CooS and two archaeal CdhAB-type CODHs [9–11]. The CdhAB-2 combines with acetyl-CoA synthase (ACS) [10] and operates in the acetyl-CoA pathway for complete oxidation of lactate to CO_2_ [12, 13]. Recently,* cdhAB-1* was shown to be transcriptionally induced in cultures using thiosulfate as an electron acceptor [13]. Which of these CODHs that are essential in the CO metabolism of* A. fulgidus* remains unknown.

In SRP, three models have been suggested for proton translocation: H_2_ cycling, formate cycling, and through “Mitchell-type” loops facilitated by respiratory menaquinone [2, 14, 15]. No H_2_ was detected in cultures of* A. fulgidus* supplemented with CO and sulfate [2] ruling out H_2_ cycling as a relevant energy conservation mechanism. On the other hand, transient formate formation was observed in cultures grown on CO and sulfate [2], but the enzymatic rationale supporting formate cycling as a mechanism for energy conservation is still lacking. A menaquinone mediated proton translocation by the F_420_H_2_:quinone oxidoreductase complex (Fqo) is crucial in energy conservation in* A. fulgidus* during growth with lactate [13]. Fqo can be hypothesized to be operative during sulfate reduction with CO. The Fqo complex receives electrons from the reduced coenzyme F_420_ (F_420_H_2_), generated from the oxidative acetyl-CoA pathway, and transfers electrons to the membrane-bound respiratory chain by the reduction of menaquinone [16–18]. The redox potential of oxidation of CO to CO_2_ (Δ*E*°′ −520 mV) is sufficient for direct reduction of ferredoxin (Fd) (Δ*E*°′ −500 mV) [19]. Whether the Fd_red_ produced in the CODH reaction may be a viable electron donor to the Fqo complex remains unknown. Besides Fqo,* A. fulgidus* harbors the quinone-interacting membrane-bound oxidoreductase (QmoABC) complex and the DsrMKJOP [20] which are highly conserved in SRP [21]. Both complexes may facilitate proton translocation in SRP, coupled to the MQH_2_ dehydrogenase:APS reductase (Qmo) and MQH_2_ oxidase:Hdr (HdrDE/DsrMK); however, the reactions are endergonic and may not readily proceed [19, 22]. Therefore, a “confurication” reaction was recently suggested for Qmo in* Desulfovibrio* [23]. This reaction is thought to involve a cytoplasmic low potential electron donor that could contribute energy to drive the oxidation of menaquinol and proton translocation through the QmoABC subunits. The Fd_red_ produced in the CODH reaction during growth with CO in* A. fulgidus* may represent a viable electron donor providing energy to drive this confurication reaction.

This study was conducted in order to facilitate a more comprehensive overview of energy conservation mechanisms in* A. fulgidus*. We performed whole genome transcriptome microarray analyses of* A. fulgidus* cultivated on CO with sulfate or thiosulfate and on CO without an electron acceptor. The results of transcriptional analysis highlight CdhAB-2 as a potential dominant CODH during growth with CO. We argue that menaquinone facilitated proton translocation by Fqo is crucial for growth with CO and is universal in* A. fulgidus* during growth with an electron acceptor (sulfate/thiosulfate). Furthermore, the Fd_red_ produced in the CODH reaction probably donates electrons directly to the Fqo complex, but apparently not to Qmo. Rather, transcripts of a nitrate reductase-associated respiratory complex were upregulated during growth with sulfate. This complex may receive electrons from Fd_red_ and cofacilitate the reduction of APS. Expression of multiple DsrMK complexes indicates an attenuated role of the DsrMKJOP complex in proton translocation in* A. fulgidus* and indicates that multiple electron-flow pathways to DsrC are facilitated. On the basis of transcriptional upregulation by CO, we propose that CooF-NoxA may form a CO specific mechanism of potential oxygen removal.

## 2. Materials and Methods

### 2.1. Strains and Cultivation

*Archaeoglobus fulgidus* strain VC16 (DSMZ 4302),* A. profundus* [24],* A. veneficus* [25], and* A. sulfaticallidus* [26] were obtained from the Deutsche Sammlung von Mikroorganismen und Zellkulturen (Braunschweig, Germany). All cultivation was performed in tubes containing 10 mL solution at 80°C under anoxic conditions, utilizing carbonate buffered media, pH 6.8, as described previously [13]. The atmosphere was pressurized to 250 kPa and consisted of CO : CO_2_ at a 80 : 20 ratio. Cultivation with various electron acceptors differed as follows: cultures with sulfate (S-CO), 2.2 g/L Na_2_SO_4_, and 3.7 g/L MgSO_4_·7H_2_O; cultures with thiosulfate (T-CO), 3.7 g/L MgCl_2_·6H_2_O and 7.45 g/L Na_2_S_2_O_3_·5H_2_O; cultures without electron acceptor (Ø-CO), 3.7 g/L MgCl_2_·6H_2_O.

*A. veneficus*,* A. profundus*, and* A. sulfaticallidus* were cultivated as described above with thiosulfate and N_2_/CO_2_ (80 : 20) atmosphere, with the following modifications:* A. veneficus*, 80°C, 30 mM pyruvate;* A. profundus*, 80°C with lactate under an atmosphere of 250 kPa H_2_/CO_2_ (80 : 20);* A. sulfaticallidus*, 75°C, 35 mM d/l-lactate. Cultures were used as inoculum in tubes containing thiosulfate and CO/CO_2_ (80 : 20) at atmospheric pressure or 25 kPa and incubated at the temperature corresponding to the strain.

Growth rates of* A. fulgidus* were monitored by turbidity measurement of optical density (OD) (Abs 600 nm). Linearity of cell numbers and OD was confirmed using direct cell counts (Thoma Chamber, depth 0.02 mm). As methylene blue-based assays are inhibited by thiosulfate [27], the concentration of sulfide was measured using the Cord-Ruwisch assay [28]. All experiments were performed on cultures adapted to their corresponding growth conditions and transferred at least 3 times prior to analysis.

### 2.2. RNA Extraction and Transcriptome Analyses

**Figure 1:**
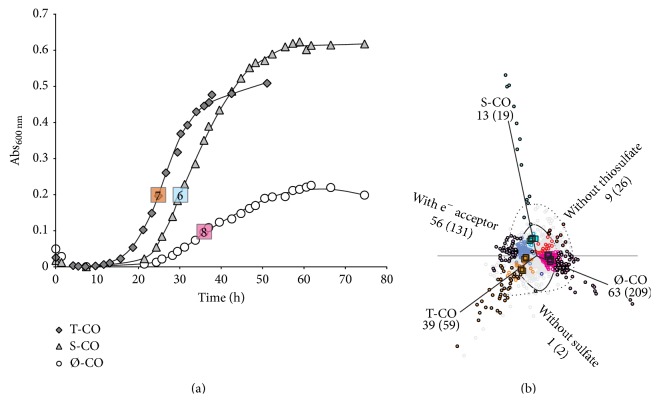
(a) Growth curves of cultures utilizing CO/CO_2_ grown on either sulfate (S-CO), thiosulfate (T-CO), or without electron acceptor (Ø-CO). Numbers denote biological replicates for each growth condition. (b) A correspondence plot (PCA 1 and 2; 24.8% and 14.6% of total variance) of differentially regulated genes (circles) displaying significantly differentially regulated genes (ANOVA) in relation to growth conditions (total amount of differentially regulated genes in brackets). Filled circles and numbers outside brackets denote differentially expressed genes above 1.5-fold. Outlined area shows differential expression above 1.5- and 2-fold.

The cultures were cooled and fixed at a predetermined absorbance corresponding to midlogarithmic growth phase (Figure 1). RNA was extracted from 2 (S-CO, T-CO) or 4 (Ø-CO) tubes in order to obtain sufficient yield (>1 *μ*g RNA). RNA extraction and all subsequent steps, including cDNA synthesis, hybridization, and image processing were performed as described previously [13]. A subset of samples were randomized and cohybridized on 4-plex arrays of the previous experiment. This was done in order to eliminate batch effects for the estimation of differential expression related to growth on CO. Quantile normalization was performed on all data, which allows the comparison of relative and absolute expression. The microarray data are available in the ArrayExpress database (http://www.ebi.ac.uk/arrayexpress/) under Accession number E-MTAB-3035. The processed data deposited for this experiment are directly comparable with those of the previous study [13], E-MTAB-2294. In this study, we report absolute abundance as a ratio from the mean value (1.0) of array expression derived from quantile normalization.

In total, 21 hybridizations were performed and were allotted correspondingly to each growth condition: 6 S-CO, 7 T-CO, and 8 Ø-CO. In order to perform ANOVA analysis, 6 values corresponding to the minimum residual sum of squares were selected per gene, per growth condition. Differential expression was identified as significant when the ANOVA returns a *p* value of less than 0.00001 (critical *F* > 37.71). Correspondence analysis was used to evaluate the ANOVA analysis [29]. As Ø-CO is the only condition that may sustain growth without an electron acceptor, evaluation of differential regulation in relation to CO was performed by S-CO and T-CO conditions versus data of the previous study. Functional annotation was performed using the archaeal clusters of orthologous genes (arCOG) and corresponding COG functional categories [30]. Functional enrichment analysis was performed on all significantly regulated genes by the Chi-squared test (*p* < 0.05 for groups larger than 5 genes). In addition to evaluation of significance by ANOVA, a fold change of 1.5 or larger was generally considered a cutoff for the evaluation of differentially regulated genes.

### 2.3. Quantitative Real Time PCR (qPCR)

Triplicate biological replicates were analyzed by qPCR on the following conditions: sulfate and lactate (S-L), thiosulfate and lactate (T-L), and thiosulfate with H_2_ and CO_2_ (T-H_2_) and Ø-CO, S-CO, and T-CO. RNA extraction was performed as described in the previous section. The cDNA synthesis was performed using the High Capacity RNA-to-cDNA Kit (Applied Biosystems) by the protocol of the supplier. Transcripts were quantified by TaqMan qPCR on a StepOnePlus Real Time PCR System (Applied Biosystems, Foster City, CA, USA). Primers and probes (Table S4, available online at http://dx.doi.org/10.1155/2015/235384) were designed for use in a standard TaqMan amplification protocol. Amplification was carried out in a final volume of 20 *μ*L containing 900 nM of each primer, 250 nM of the probe, 1x TaqMan Gene Expression Master Mix (Applied Biosystems), and 1 *μ*L of cDNA. The reactions were performed under the following conditions: 2 min at 50°C, 10 min at 95°C, then 15 sec at 95°C, and 1 min at 60°C, respectively, for 40 cycles. Triplicate biological replicates were analyzed. The gene AF0424 (*dsrB*) was used as endogenous control. Variance of AF0424 between samples was consistently <1 cycle threshold (Ct) supporting the use as endogenous control. This result is corroborated by results from the microarray analyses. Background levels of endogenous DNA were consistently above a shift in 20 Ct's (ΔCt) beyond the endogenous control.

### 2.4. Bioinformatic Tools

Bioinformatic tools were used to evaluate the following: homology, BLASTp and PSI-BLAST; functional domains, Conserved Domains Database (CDD) (http://www.ncbi.nlm.nih.gov/guide/all/#tools); transmembrane segments, TMHMM Server v. 2.0; signal peptide prediction, SignalP 4.1 Server (http://www.cbs.dtu.dk/services/); inter- and intragenomic synteny, STRING database (http://string-db.org/), and Absynte and Syntax tools (http://archaea.u-psud.fr/).

## 3. Results

### 3.1. Growth with CO

Final concentrations of sulfide were equivalent in S-CO and T-CO cultures (~8-9 mM), and a decrease in pH was observed during all conditions being consistent with acetate production during growth with CO and sulfate [2]. Growth rates increased significantly when thiosulfate was present (Figure 1(a); *t*-test, *p* < 0.05; T-CO, 1.31 ± 0.57 h doubling time). Lower growth rates were observed for cultures during growth with S-CO (2.41 ± 0.25 h) and during Ø-CO (2.80 ± 0.42 h). The observed growth rates on CO were comparable to growth with lactate, as previously reported [2, 13].

During a period of 2 months, neither an increase in turbidity increase nor sulfide production was observed in 10 replicate cultures of* A. veneficus*,* A. profundus*, and* A. sulfaticallidus*, respectively. Cultivation was attempted under CO/CO_2_ at atmospheric pressure or 250 kPa with their common electron acceptor thiosulfate. Hence, CO does not seem to be a viable growth substrate of these species in the genus* Archaeoglobus*.

### 3.2. Transcription Profile of Growth with CO

When CO was utilized as a substrate, 52 genes (36 up/16 down) were uniquely differentially regulated by more than 1.5-fold (Figure S1). This was the result of comparing transcriptional profiles of CO growth with electron acceptor (S-CO, T-CO) to previous reported data on lactate and hydrogen, with electron acceptors, sulfate, or thiosulfate [13].

The COG category inorganic ion transport (P) was the category most enriched in presence of CO (Table S1). Of the highly differentially regulated transcripts (>1.5 fold), the most highly upregulated gene cluster encoded a putative phosphate ABC transporter (AF1356–AF1361). These were upregulated in both S-CO and T-CO cultures but not in Ø-CO. Minor levels of upregulation was observed for genes in the categories DNA replication (L), translation (J), and coenzyme transport and metabolism (H), where only the gene of N-glycosylase/DNA lyase (J, AF0371) and glutamate-cysteine ligase (H, AF2307) were upregulated above 2-fold (Table S2).

The gene* cooF* (AF0950, Table S2, Figure 3(b)), which encodes a homologue of a carbon monoxide dehydrogenase binding iron-sulfur enzyme, was highly upregulated by CO conditions. In other carboxydotrophic species,* cooF* is often colocated with the carbon monoxide gene* cooS* [11]. In* A. fulgidus*, noxA*-4* encoding a NADH oxidase (AF0951) is the adjacent gene to* cooF*. These genes were coexpressed by growth with CO (Table S2).

Transcripts downregulated in the presence of CO correspond to the COG category of amino acid transport and metabolism (E, Tables S1 and S2). Two colocated genes encoding a tryptophan repressor protein, desulfoferredoxin (AF0343, AF0344), and genes encoding an aldehyde ferredoxin reductase (*aor*; AF0077, AF2281) were downregulated by more than 1.5-fold. This was also the case for the gene* feoB-1* of an iron transporter enzyme (AF0246).

**Figure 2:**
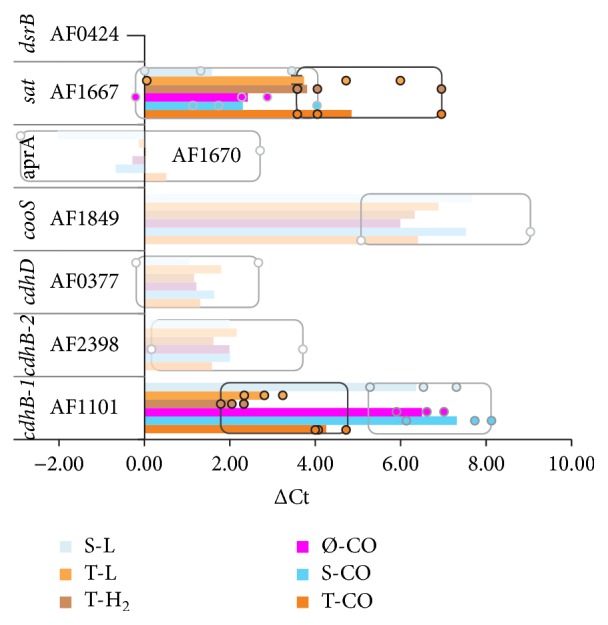
Overview of shift (ΔCt) in real time PCR cycle threshold (Ct) values between control (*dsrB*/AF0424) and subsequent assayed transcripts. Average ΔCt values are indicated by bars. The data range for nonsignificant shifts in gene expression is transparent and the data range (min-max) is indicated by boxes. A significant shift (ANOVA) was found between thiosulfate grown cultures (black squares) and cultures with sulfate, or no electron acceptor (gray squares) for AF1101 (ΔΔCt −2.22, *p*  4.7 × 10^−7^) and AF1667 (ΔΔCt 1.97, 0.04). Low expression levels (i.e., high ΔCt values) indicate lower levels for transcripts of cooS during all conditions and cdhB-1 during growth with sulfate or no electron acceptor.

**Figure 3:**
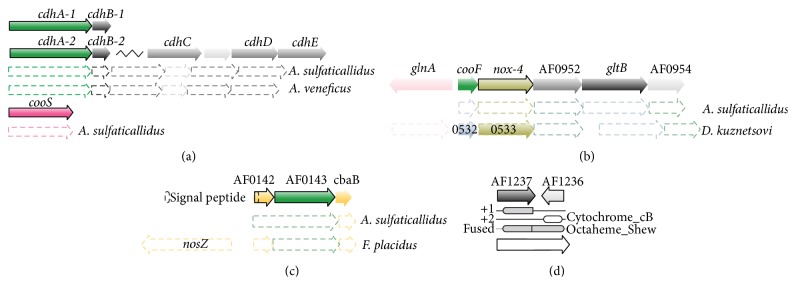
Homology and synteny of key genes: (a) genes of carbon monoxide dehydrogenase (CODH) and (b) the genes encoding CooF and associated NADH oxidase (nox-4). (c) genes of a putative nitrate reductase-like respiratory complex (AF0142–AF0144) and (d) the reading frame shift and encoded domains corresponding to a complete sequence of putative cytochrome c.

Notably, the transcripts encoding CODH's, the* CooS* (AF1849), the CdhAB-1 (AF1100, AF1101), and the CdhAB-2 (AF2397, AF2398) were not differentially expressed in the presence of CO (Figures 2 and 3, Table S2). However, the transcripts of* cdhA-2* are continuously expressed at approximately 3.6 levels above the average signal expression on microarrays. This is supported by qPCR Ct values that were comparable with expression of* dsrB*. Furthermore,* cooS* and* cdhAB-1* were expressed at lower transcriptional levels; both were expressed at average signal expression levels (1.0) during Ø-CO and S-CO conditions. While transcripts of* cdhB-1* were upregulated during T-CO conditions, upregulation of cdhB-1 was confirmed by qPCR. Low transcriptional expression of* cooC* is indicated by high positive ΔCt values (>5) (Figure 2).

### 3.3. Transcription Profile of Growth with Sulfate

**Figure 4:**
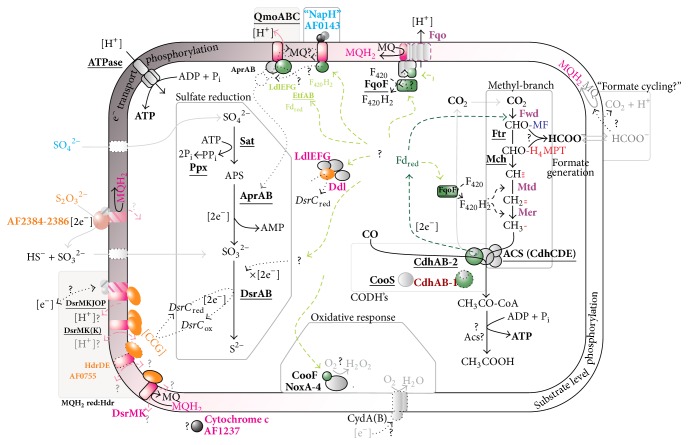
Presentation of putative mechanisms of energy conservation in* A. fulgidus* during carboxydotrophic growth. Acetate generation is probably a result of substrate level phosphorylation (right). Energy conservation is also coupled to electron transport phosphorylation (left) when electron acceptors sulfate (SO_4_^2−^) or thiosulfate is present (S_2_O_3_^2−^). Dotted lines indicate pathways of electron flow. Probable or putative paths of electron transport from Fd_red_ to the terminal electron acceptor are shown in green or light green, respectively. Names of gene products are highlighted accordingly: in bold: expressed at or upregulated to high signal intensity (>3); underlined: constitutively expressed. The colors indicate the condition corresponding to upregulation (as in Figure 1(b)): blue: S-CO; orange: T-CO; magenta: Ø-CO. Downregulated genes during Ø-CO: purple (for data, see Table S2 in Supplementary Material). Cofactors of the methyl-branch: methanophenazine (MF), 5,6,7,8-tetrahydromethanopterin (H_4_MPT).

During S-CO conditions, 13 transcripts were identified as upregulated above 1.5-fold change (Figure 1(b)), while, only one, encoding a uncharacterized conserved protein (AF1464, Table S2) was downregulated. The low number of differential expression during S-CO growth conditions eliminated the need for a broader functional characterization (COG). The highly upregulated transcripts encode a cytochrome c oxidase subunit II and a periplasmic nitrate reductase (Nap) associated protein (AF0141–AF0144 and AF0189-AF0190, Figures 3(c) and 4, Table S2). In silico analysis provided the basis for further elucidation. The gene AF0143 is a homologue of* napH*, which encodes a putative quinone-interacting membrane-bound nitrate reductase-associated enzyme [31]. The AF0143 encodes a putative protein with 9 transmembrane regions containing multiple ferredoxin ([4Fe4S]) binding sites (TMHMM Server, CDD). This gene is flanked by two genes encoding the Cu-binding subunit II of cytochrome c oxidase (AF0142, AF0144, and CDD). The AF0190 transcript increased 4-fold, but expression levels were relatively low (Table S2). The AF0190 gene encodes yet another domain of cytochrome c subunit II, the cytochrome_CBB3 (Pfam, CDD), haem-binding domain. The genes, AF0143 and AF0144, are conserved in related genera* A. sulfaticallidus* and in* Ferroglobus placidus* (Figure 3(c)). In* F. placidus,* these genes are adjacent to a nitrous oxide reductase (*nosZ*/Ferp_0128) [32].

### 3.4. Transcription Profile of Growth with Thiosulfate

Transcriptional shifts by T-CO corresponded largely to previously identified regulation corresponding to thiosulfate utilization [13]; these transcripts encode a putative periplasmic thiosulfate reductase (AF2384–AF2386, Figure 3); the CdhAB-1 (AF1100-AF1101, Figures 2 and 4, Table  S2); and an iron ABC transporter (AF0430–AF0432) (Table S2). Furthermore, the transcripts encoding a membrane-bound heterodisulfide reductase (AF0755) homologue of the DsrMK complex was induced 3-fold (Figure 4).

In addition, a gene cluster adjacent to the putative periplasmic thiosulfate reductase was upregulated to high expression levels (AF2380–AF2383, Table S2). These genes are homologs of a conserved gene cluster in* Desulfovibrio* that may control cellular morphology [33, 34]. They also encode subunits of a putative cobyrinic acid a,c-diamide synthetase (CbiA) (AF2380, AF2383). Moreover, transcripts of putative glutaredoxin and ferredoxin-thioredoxin reductase (FTR) (AF1534–AF1536) were induced by more than 2-fold [35], suggesting function in an unknown regulatory system. Finally, upregulation by a high-fold change, but at low levels of absolute abundance, was found for transcripts encoding a putative iron ABC transporter (AF0429–AF0433) and an ABC transporter with unknown specificity (AF1981–AF1984). Similar transcriptional profile was shared by molybdopterin oxidoreductase that may also function as thiosulfate reductase (AF0154–AF0157, ~0.2 level of average transcriptional expression, Table S2).

The most highly downregulated transcripts during T-CO conditions were of a branched-chain amino acid ABC transporter (AF0221, Table S2).

### 3.5. Transcription Profiles of Acetogenic Growth without an Electron Acceptor

Growth in the absence of electron acceptor (Ø-CO) caused the most extensive transcriptional response, where 119 genes were differentially regulated above 1.5-fold (5.0% of total genes, 63/56; up/down). The genes were in the COG categories translation (J), replication of DNA (L), defense (V), metabolic processes of nucleotide (F), and carbohydrate (G) (Table S1). However, little clear metabolic information could be extracted from this broad functional enrichment. In the COG category defense (V), the genes of ATP-dependent RNA helicase (AF0071), CRISPR-associated genes (cas4, cas1, and* recB*: AF1877–AF1879, Table S2) were highly upregulated. Except for transcripts of ribosome biogenesis (J; AF0058 and AF0734) and a ribonuclease M5 (L; AF0905) (Table S2), the category of “translation and replication of DNA” was not induced above 1.5-fold. This is also true for transcripts encoding nucleotide and carbohydrate metabolic processes (<1.5-fold).

During Ø-CO conditions, genes encoding the following three putative operons encoding potential membrane-bound redox enzymes were upregulated (Table S2, Figure 3): the* dsrMK* homolog (AF0546-AF0547); a partial homologue of membrane-bound tetraheme cytochrome c subunit (AF1237-AF1236); and a putative multimeric d/l-lactate dehydrogenase (AF0808–AF0811).

The nucleotide sequence of the region containing the membrane-bound tetraheme cytochrome c subunit (AF1237) corresponds to a larger gene encoding a single cytochrome c homologue that was upregulated during Ø-CO conditions (Figure 3(d)). Reinvestigation of the genetic region found a sequence that probably constitutes a single reading frame of a complete cytochrome c, containing the Pfam domains “octaheme_Shew” (AF1237) and adjacent region, on the reverse strand of AF1236, encoding a “Cytochrome_cB” domain, where the latter domain is a shifted one frame position. This differential transcription may be best explained by adaptions to absence or low levels of electron acceptor, indicating putative high affinity complexes. Transcripts of an octaheme homologue in* D. vulgaris* strain Hildenborough (DVU3144) are upregulated during growth with limiting concentrations of sulfate [36]. These results were linked to an increase in hydrogen cycling in* D. vulgaris*. However, the cytochrome c in* A. fulgidus* probably does not facilitate a similar mechanism, as it lacks many homologues of the membrane-bound and periplasmic complexes of* D. vulgaris* [21].

Transcripts encoding genes of putative multimeric d/l-lactate dehydrogenase (AF0808–AF0811) were upregulated by more than 1.8-fold during growth on Ø-CO. These genes were previously shown to be specifically upregulated by growth with sulfate and lactate [13]. The upregulation in relation to growth with CO and specifically on Ø-CO indicates an additional unknown role in energy metabolism (Figure 4). In this respect, it is interesting to note that unadapted cells adapted more slowly to growth on CO in the presence of lactate, as observed previously by Henstra et al. [2].

Finally, two clusters of genes encoding uncharacterized proteins (AF0407-AF0408 and AF1575-AF1576, Table S2) were highly upregulated during acetogenic growth (Ø-CO). The gene AF0407 corresponds to an unknown conserved domain sequence within archaea (GOG1772 or DUF531) that may correspond to a S-adenosyl-L-methionine- (SAM-) dependent methyltransferase [37]. The upregulation of these transcripts indicates that methylation functions in an important, but unknown, regulatory or biosynthetic role during Ø-CO conditions.

Downregulation in the absence of electron acceptor (Ø-CO) corresponds to the categories of energy (C), carbohydrate (G), lipid (I), and inorganic ion (P) transport and metabolism (Table S1). Transcripts of energy metabolism (C) downregulated by more than 1.5-fold encode ferredoxin (*fdx-3*, AF0355) and a d-lactate dehydrogenase (AF0394) (Figure 4, Table S2). Minor downregulation was observed for genes encoding enzymes catalyzing the acetyl-CoA pathway: the methylenetetrahydromethanopterin reductase (*mer-1*) and tungsten formylmethanofuran dehydrogenase, subunit B (*fwdB-1*) (Figure 4, Table S2). Fumarate dehydrogenase (*fum-1* and* fum-2*) of the anabolic TCA cycle was also downregulated. Also, the genes AF1823 and AF1826 encoding subunits of the membrane-integrated components of respiratory complex I homolog (*fqo)* [18] were downregulated (Figure 4, Table S2).

In the category carbohydrate metabolic processes and transport (G, Tables S1 and S2), genes encoding a carbohydrate kinase (AF0401), a phosphomannomutase (AF0458), as well as putative transporters of hexuronate and drug/metabolites (AF0013 and AF0787-AF0788), were highly downregulated. This was also the case for genes in the category fatty acid metabolism (I) encoding: enoyl-CoA hydratase (AF0963), acyl-CoA dehydrogenase (AF0964, AF2244), acetyl-CoA acetyltransferase (AF0967, AF2243), and acetyl-CoA synthetase (AF1287). Other upregulated transcripts encode enzymes involved in oxidation of uneven fatty acids/propionate metabolism, the putative methylmalonyl mutases (AF1288a, -b and AF2219). Finally, high-fold downregulation of genes in the category inorganic ion transport (P, Tables S1 and S2) encoded putative transporters of iron (*feoB-1*; AF0246), Ca^2+^/Na^+^ antiporter (AF0251), and a phosphate ABC transporter (AF1356–AF1359).

Notably, genes encoding the canonical sulfate reduction pathway were expressed at high transcriptional levels (>3 times average expression) during all growth conditions. In relation to growth with thiosulfate versus sulfate and absence of electron acceptor (S-, Ø-) a significant shift in transcriptional expression of* sat* was identified by qPCR (Figure 2), but it was not confirmed by microarray analysis (Table  S2). This is in agreement with the previously observed hydrogen-influenced downregulation of genes related to sulfate/APS reduction, rather than the absence of sulfate as electron acceptor [13].

## 4. Discussion

By the use of transcriptome profiling, this study identifies enzymes and redox components in* A. fulgidus* that are putatively involved in growth with CO. Distinct responses to sulfate (S-CO) or thiosulfate (T-CO), and growth without a terminal electron acceptor (Ø-CO), were identified. A conceptual model of the CO metabolism of* A. fulgidus* is presented in Figure 4.

### 4.1. Electron Transport Phosphorylation

We propose that the bifunctional ACS/CODH is involved in the primary oxidation of CO in* A. fulgidus*, due to the constitutive expression of genes encoding the enzyme complex. In contrast, the monofunctional CODH (CooS) appears to play a smaller role in CO metabolism. Transcripts of* cooS* was detected at average transcriptional levels on microarrays (~1.0) and is detected at relatively high ΔCt values during qPCR (Figure 2). Previously 2-fold upregulation of* cooS* was identified during late-log sulfate and lactate (S-L) conditions [13], making the link between* cooS* and CO metabolism less clear. On the basis of transcriptional expression and differential regulation, it is probable that CdhAB-2 can catalyze both CO oxidation and CO assimilation (Figure 4). That CO utilization requires only one* cdhAB* isoform is consistent with previous studies of* Methanosarcina acetivorans* [38, 39].

Apparently, a general mechanism for thiosulfate utilization, specifically involving a putative periplasmic thiosulfate reductase (AF2384–AF2386, Figure 4) is in place in* A. fulgidus*, as described previously [13]. We reconfirm that the transcriptional upregulation of* cdhAB-2* is tightly connected with the presence of thiosulfate (Figure 2 and Table S2). Minor upregulation of transcripts encoding molybdopterin oxidoreductase (AF0157–AF0159) may indicate an additional thiosulfate reductase.

We have previously questioned whether Fd_red_ donate electrons directly to Qmo and facilitate a “confurcation” mechanism due to an impasse related to bifurcation during hydrogenotrophic growth in* A. fulgidus* and* A. sulfaticallidus* [13]. In the presence of sulfate, transcripts encoding a novel nitrate reductase-like respiratory complex were upregulated (AF0141–AF0144, Section 3.3 of this paper). In silico analysis revealed that AF0143 encodes a transmembrane complex with cytoplasmic facing [4Fe4S] domains which may facilitate interaction with Fd_red_. The gene AF0143 encodes a NapH homologue which is an integral membrane component of nitrate reductase. Despite the presence of homologous genes [9], no species of Archaeoglobales are capable of nitrate respiration [26]. By inferences from denitrifying bacteria, the NapH homologue can assign the ability to ferry electrons from cytoplasm to the periplasm [40, 41] and facilitate redox interactions with menaquinone [31, 42]. Association with the MQ pool seems plausible, but we cannot determine whether this complex may facilitate oxidation or reduction of MQ/MQH_2_. The Qmo complex and its subunits are not ubiquitous amongst the SRP [21]. However, due to the constitutive expression of* qmoABC* and lack of putative FAD/FMN binding domains in “NapH” questions that the nitrate reductase like complex replaces Qmo. Rather, this complex may function in receiving electrons from Fd_red_. The electrons from cytoplasmic oxidation of CO may thus flow from the nitrate reductase-like respiratory complex via menaquinone to Qmo, forming a dedicated redox couple (Figure 4). However, this mechanism does not clearly contribute to an increased understanding in the role of Fd_red_ regarding the energetic problems of proton translocation coupled to APS reduction [23]. Hence, there is a clear need for future biochemical studies to evaluate the potential associations between APS reduction, QmoABC, and Fd_red_ and to define a role for the nitrate reductase-like complex in the respiratory chain of* A. fulgidus*.

Of 8 ferredoxins (*fdx*), only the* fdx-3* (AF0355) was downregulated during growth with CO without an electron acceptor (Table S2). The gene AF0355 may therefore encode a respiratory ferredoxin. As all genes encoding the Fqo complex were highly expressed when thiosulfate and sulfate were added in the growth media (Table S2), our model suggests that this complex plays an important role in generating proton translocation and menaquinone reduction in the presence of a terminal electron acceptor (Figure 4). This implies that Fqo complex facilitates menaquinone reduction via a Fd_red_:(mena)quinone oxidoreductase reaction. Likewise, in CO-grown* M. acetivorans,* the homologous Fpo complex facilitates proton expulsion and reduction of methanophenazine, ultimately resulting in reduction of the heterodisulfide bonds forming between coenzymes M and B (CoM-S-S-CoB) in the final methane-forming step in the methanogenesis [43, 44].

The data furthermore revealed that the genes encoding DsrMKJOP and DsrMKK (AF0543–AF0545) are constitutively expressed, as seen previously [13], and genes encoding a fused DsrMK (AF0755) were upregulated by minor levels in response to thiosulfate. The substrate of DsrK is most probably C-terminal cysteine bonds in DsrC [45, 46]. DsrC is believed to be an electron transfer protein capable of ferrying electrons from menaquinol reduction to DsrAB for terminal reduction of sulfite [46] (Figure 4). Genes encoding a third DsrMK complex (AF546-AF547) were uniquely transcribed in the absence of terminal electron acceptor (Ø-CO). This indicates that transcripts of this complex are upregulated to facilitate low levels of electron flow to DsrC in order to reduce trace amounts of external electron acceptors.

The Fpo complex of* M. mazei* Gö1 is able to translocate approximately 2 protons coupled to the reduction of methanophenazine (MP; *E*°′_MP/MPH_2__ −170 mV) [47]; it is therefore possible that the Fqo complex in* A. fulgidus* translocates 4 protons coupled to reduction of the higher potential electron acceptor MQ (*E*°′_MQ/MQH_2__ −80 mV). This indicates that the Fqo complex alone may be the major mechanism of energy conservation in* A. fulgidus* [19]. The presence of genes encoding multiple DsrMK in addition to the DsrMKJOP complex as found in* A. fulgidus* is infrequent among the SRP [21]. Energy conservation by Fqo is dependent on the reoxidation of menaquinol (MQH_2_) which may be facilitated by the complete DsrMKJOP and by the heterodisulfide reductase (DsrMK) complexes (Figure 4).

It is possible that multiple DsrMK complexes may couple electron flow to DsrC via proton-translocating coupled oxidation of MQH_2_ [22], but it is also possible that some DsrMK complexes may facilitate non-proton-translocating oxidation of MQH_2_, facing the intracellular space (Figure 4). Both the non-energy- and energy-conserving reactions are so far considered thermodynamically unfavorable due to the high redox potential of sulfite (*E*°′ −116 mV) [19, 22]. However, with less energy input, a non-energy-conserving MQH_2_:DsrC oxidoreductase could facilitate electron flow and energy conservation by the Fqo complex, coupled to terminal reduction of sulfite. This may indicate the potential for attenuated roles of DsrMKJOP in the presence of multiple DsrMK complexes. However, until a mechanism of MQH_2_ oxidation is more clearly characterized within the SRP, this cannot yet be considered a viable model.

### 4.2. Substrate Level Phosphorylation (SLP)

Acetate and transient levels of formate form together with sulfide when* A. fulgidus* is cultivated with CO and sulfate, [2]. During growth with CO only, acetate accumulates with increased rates of transient formate [2]. Consistent with this reported acetate formation, a decrease in pH was observed during all CO growth conditions. Formation of acetate suggests that ATP is generated by SLP from acetyl-CoA formed via the reductive acetyl-CoA pathway [2]. Interestingly,* A. fulgidus* lacks genes encoding an acetate kinase (Ack) and a phosphotransacetylase (Pta) [9] which catalyze SLP and concomitant acetate formation during carboxydotrophy in* M. acetivorans* [43, 44]. A viable option for* A. fulgidus* may be found in studies of* Thermococcus onnurineus* [48]. Here, substrate level phosphorylation and acetate formation from acetyl-CoA may be catalyzed by acetyl-CoA synthetase (Acs). In* A. fulgidus* however, no upregulation of the 6 acetyl-CoA synthetase (Acs) encoded in the genome [9] was observed, nor were these genes transcribed at high levels (Table S3). Therefore, rather surprisingly, our study does not provide any clear information about putative enzymes responsible for acetate formation and energy conservation by SLP (Figure 4).

### 4.3. Generation of Reduced Factor 420 (F_420_) and Transient Formate Generation

Reduction of CO_2_ to methyl in the acetyl-CoA pathway requires Fd_red_ in the initial formyl-generating step, while the cofactor F_420_H_2_ is utilized by the methylene and methyl generating reactions (Figure 4). However, in the absence of a F_420_ reducing [NiFe] hydrogenase (Frh) found in most methanogens,* A. fulgidus* lacks a clear mechanism for the generation of F_420_H_2_ [49]. Previously, Welte and Deppenmeier have confirmed that the soluble FpoF subunit of the Fpo complex independently catalyzes the reduction of F_420_ with Fd_red_ in* M. mazei* [50]. The homologues FqoF complex also contains FAD and acid liable iron-sulfur clusters [18]. This can indicate a potential of the Fqo complex to link Fd_red_ to both F_420_ reduction and energy conservation (MQ) by flavin dependent bifurcation. However, it is shown that Fpo complex in* Methanosaeta thermophila* is capable of catalyzing aFd:MP oxidoreductase reaction independent of the FpoF subunit [51]. Consistent with the absence of electron acceptor (Ø-CO), transcripts of* fqo* genes encoding membrane-bound subunits are downregulated (Figure 4). In* A. fulgidus,* the observed constitutive expression of* fqoF* (AF1833) is in support of F_420_H_2_ generation from Fd_red_ by the FqoF subunit, albeit the continued association between FqoF and Fqo complex cannot be known (Figure 4).

When* A. fulgidus* is cultivated on CO in the absence of electron acceptor, acetate generation from acetyl-CoA via SLP is probably the major energy conservation [2], though the exact mechanism is unknown. It was therefore surprising to note that genes of the acetyl-CoA pathway were downregulated under this growth condition (Ø-CO). These results may seem counterintuitive as they indicate less expression of the pathway of reductive assimilation of CO_2_. This, in turn, would result in reduced rates of acetyl-CoA generation and subsequent ATP production by SLP. However, these results could explain the observed increase in transient formate during growth on CO without an electron acceptor [2]. Formate may be generated from a formyl group from the initial reductive steps of the acetyl-CoA pathway (formyl-methanofuran or formyl-tetrahydromethanopterin, Figure 4); the downregulation of F_420_H_2_ dependent steps of the acetyl-CoA pathway may support this observation. If the Fd_red_ dependent rate of reduction CO_2_ exceeds the rate of Fd_red_ dependent F_420_H_2_ formation, the availability of F_420_H_2_ would limit the rate of reduction of formyl to methyl. This could facilitate hydrolysis of the formyl intermediate to formate.

The generated formate may exit the cell and be reoxidized in the extracellular space. However, we did not observe any differential expression of the putative formate dehydrogenase (AF1203, AF1202) in* A. fulgidus* or any data supporting formate cycling as a mechanism of proton translocation, nor did we observe any expression of the two hydrogenases Vht and Mvh:Hdl [52] which are expressed during growth on hydrogen [13]. It is possible that formate dehydrogenase is induced during late-log growth as these conditions were not assayed. However, in the presence of sulfate or thiosulfate, it may be expected that energy conservation would be facilitated by the cytoplasmic generation of formate and subsequent periplasmic oxidation by a “formate cycling” mechanism [2, 15]. We have not identified transcriptional regulation that supports the presence of such mechanism. On this basis, it seems that formate reenters the acetyl-CoA pathway by an unknown condensation reaction during late log-growth (Figure 4).

### 4.4. Adaptation to CO and a Potential CO Specific Oxygen Removal

In this experiment,* A. fulgidus* was grown in a 80% CO atmosphere at 250 kPa pressure. Generally, CO inhibits dissimilatory sulfate reduction in some SRP at concentrations as low as 2% CO [3]. The Deltaproteobacteria are generally inhibited by concentrations up to 20% CO, while the generality of this may be demonstrated by inhibition of SRP in bioreactor experiments at similar levels [3]. This CO inhibition has been attributed to interactions of CO with metalloenzymes, including hydrogenases [3]. In contrast,* Desulfotomaculum* sp. may tolerate up to 50% CO [3] and the carboxydotrophic* D. carboxydivorans* CO-1SRB grows in a 100% CO atmosphere [53]. Archaeal and clostridial SRP maintain few genes for periplasmic cytochromes c and the associated redox complexes present in deltaproteobacterial SRP [21]. Therefore, energy conservation facilitated by respiratory menaquinone in “Mitchell-type” loops may serve as an explanation for the high CO tolerance in* A. fulgidus*. Consistently, our data did not reveal any novel resistance complexes and the regular pathways of dissimilatory sulfate and sulfite reduction seem capable of functioning at high partial pressures of CO.

The only transcript of discernable function universally upregulated in the presence CO is* cooF* (AF0950, Figures 3(b) and 4). The CooF is suggested to take part in electron transfer from CO oxidation by CooS [11, 54]. In* A. fulgidus,* no synteny is observed between these two genes (cooS; AF1849) although they are commonly colocated in other carboxidotrophs [11]. In* A. fulgidus*, the* cooF* (AF0950) is colocated with a gene of NADH oxidase (*noxA-4*) and an adjacent conserved gene cluster encoding a glutamate synthetase (AF0952–AF0954, Figure 3(b)). However, only the genes* cooF* and* noxA-4* were transcriptionally upregulated by growth with CO. The NADH oxidase (NoxA) has a potential role in oxygen removal, by catalyzing the reduction of O_2_ to H_2_O_2_ [55]. Therefore, the genes* cooF* and* noxA-4* may encode a mechanism of CO specific oxygen removal in* A. fulgidus* (Figure 4). These genes are conserved in the acetogenic carboxydotrophic* D. kuznetsovii* [49]. However, a similar genomic organization is found in a wide range of bacteria with no clear link to CO (Absynte server search, seeded with AF0950). Given this conserved synteny, further experiments are needed to validate the role of these enzymes.

### 4.5. Carboxydotrophy within the Genus Archaeoglobus

Altogether, our results suggest that carboxydotrophic growth in* A. fulgidus* can be considered almost an intrinsic capacity, with little need for induction of transcripts corresponding to genes of metabolism, respiration, or resistance. With the exception of* A. profundus* which lacks CODHs [49, 56], it was surprising that this metabolic capacity is restricted to* A. fulgidus*. While,* A. veneficus* lacks* cooS*, it retains* cdhAB* and a complete acetyl-CoA pathway (Figure 3(a)). Since* CooS* does not seem crucial for with CO in* A. fulgidus*, the absence of a* cooS* should not explain the observed inability to grow with CO. However,* A. veneficus* lacks genes encoding CooF and NoxA (Figure 3(b)) and LldEFG which are transcriptionally upregulated in* A. fulgidus*. In contrast,* A. sulfaticallidus* maintains genes of* cooS*,* cdhAB*,* cooF*, and* noxA* (Figures 3(a) and 3(b)), as well as* lldEFG,* and could therefore have the metabolic capacity to grow with CO. We must therefore underline that further biochemical evidence is needed to support the novel functions proposed for the encoded complexes. In this respect, cultivation, but not genome inference, still seems the most promising method of discovering new carboxidotrophs.

## 5. Conclusion

The transcriptome analyses performed in this study of carboxydotrophic growth of* A. fulgidus* have revealed a number of new aspects of how this microorganism conserve energy and can grow under high CO. In particular, this research highlights the upregulated transcripts of a novel nitrate reductase-like complex that can be crucial in linking electron flow from Fd_red_ to APS reduction. This result, supported by our previous report [13], points to the independence of APS reduction (by Qmo) and sulfite reduction (by DsrAB) from Fd_red_ as an electron donor. Our study, further emphasizes a ubiquitous role on the F_420_H_2_:quinone oxidoreductase complex (Fqo) in promoting proton translocation and respiration also via Fd_red_:F_420_ oxidoreductase. Ultimately, this supports energy conservation through membrane-integral “Mitchell” loops in* A. fulgidus*. The FqoF subunit probably catalyzes the Fd_red_:F_420_ oxidoreductase reaction. Hypothetically, the imbalanced rates of electron flow via Fd_red_ and derived F_420_H_2_ to the acetyl-CoA pathway may explain the increase in transient formate generation during CO growth without electron acceptor [2]. Notably, the enzymes involved in acetate generation by substrate-level phosphorylation remain unidentified.

## Supplementary Material

Supplementary Figure S1: shows the results of Correspondence Analysis of differentially transcribed genes corresponding to variable growth conditions.
Supplementary Table S1: shows the distribution and enrichment of up-regulated genes and COG categories identified for the different growth conditions.
Supplementary Table S2: displays an overview of differentially transcribed genes.
Supplementary Table S3: shows transcription profiles of acetyl-CoA synthetase genes.
Supplementary Table S4: provides primers and probes used for quantitative real-time PCR.
