# Supplementary material for: Assessment of the Carbon Monoxide Metabolism of the Hyperthermophilic Sulfate-Reducing Archaeon Archaeoglobus fulgidus VC-16 by Comparative Transcriptome Analyses
Source: Archaea. 2015 Aug 6;2015:235384. doi: 10.1155/2015/235384 (PMC4543118; doi:10.1155/2015/235384)
Supplement: Supplementary file 1 — Supplementary Figure S1: shows the results of Correspondence Analysis of differentially transcribed genes corresponding to variable growth conditions. Supplementary Table S1: shows the distribution and enrichment of up-regulated genes and COG categories identified for the different growth conditions. Supplementary Table S2: displays an overview of differentially transcribed genes. Supplementary Table S3: shows transcription profiles of acetyl-CoA synthetase genes. Supplementary Table S4: provides primers and probes used for quantitative real-time PCR. [file 235384.f1.docx]

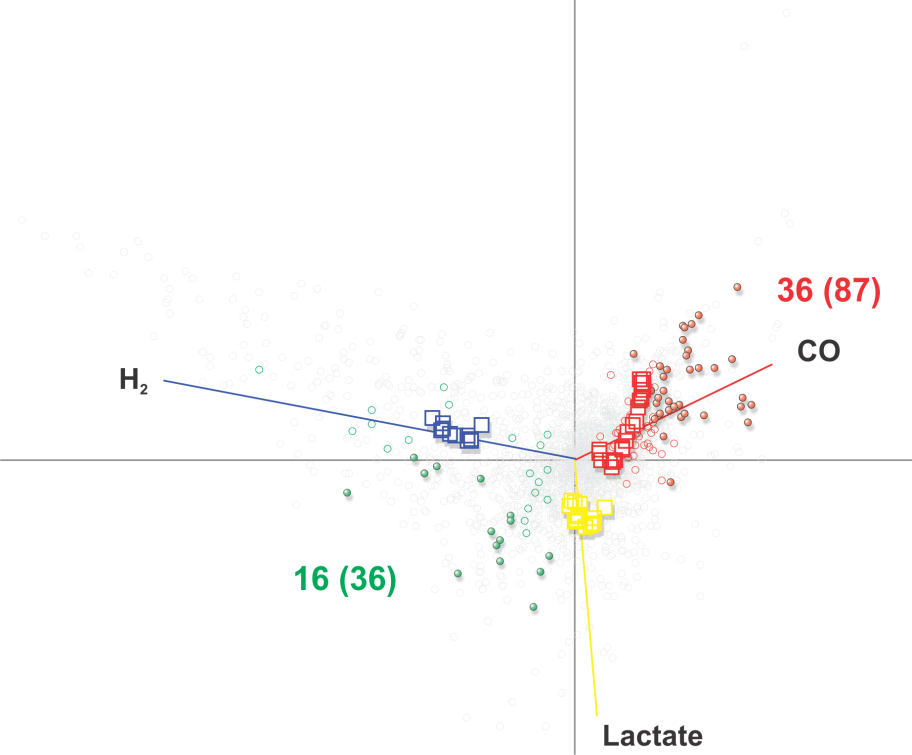


**Figure S1** Plots displaying the results of Correspondence Analysis (PCA 1 and 2; 20.0% and 10.4% of total variance) on differential regulation of genes corresponding to variable electron donors of metabolism for the current and previous study [13]. Filled circles and numbers outside brackets denote differentially expressed genes (ANOVA) above 1.5 fold.

| COG | Category | Growth condition | | | | | | | |
| --- | --- | --- | --- | --- | --- | --- | --- | --- | --- |
|  | Growth conditions | CO | w.o. CO | Ø-CO | w. e^-^ acceptor | S-CO | w.o. sulfate | T-CO | w.o thiosulf-ate |
|  | Number of diff. regulated genes | 87 | 36 | 209 | 130 | 19 | 2 | 59 | 26 |
|  | INFORMATION STORAGE AND PROCESSING | 11 | 5 | **44** | 5 | 0 | 0 | 5 | 4 |
| J | Translation; ribosomal structure and biogenesis | 0 | 0 | **20** | 2 | 0 | 0 | 1 | 2 |
| K | Transcription | 3 | 2 | 12 | 1 | 0 | 0 | (4) | 2 |
| L | Replication; recombination and repair | **8** | (3) | **12** | 2 | 0 | 0 | 0 | 0 |
|  | CELLULAR PROCESSES AND SIGNALING | 10 | 4 | 32 | 15 | 3 | 0 | 9 | 2 |
| D | Cell cycle control; cell division; chromosome partitioning | 0 | (1) | 1 | (1) | 0 | 0 | (1) | 0 |
| M | Cell wall/membrane/envelope biogenesis | (4) | 0 | 4 | **6** | (1) | 0 | 0 | 0 |
| N | Cell motility | 0 | 0 | 0 | (2) | 0 | 0 | (2) | 0 |
| O | Posttranslational modification; protein turnover; chaperones | 2 | 0 | 6 | 2 | 0 | 0 | (4) | 1 |
| T | Signal transduction mechanisms | (3) | 1 | 4 | 1 | 0 | 0 | 0 | 1 |
| U | Intracellular trafficking; secretion; and vesicular transport | 0 | 0 | 0 | (3) | 0 | 0 | (1) | 0 |
| V | Defense mechanisms | 1 | 2 | **17** | 0 | (2) | 0 | 1 | 0 |
|  | METABOLISM | 25 | 14 | 51 | **76** | 6 | (1) | **27** | 9 |
| C | Energy production and conversion | 5 | 4 | 12 | **31** | **6** | 0 | **9** | 1 |
| E | Amino acid transport and metabolism | 2 | **6** | 9 | 8 | 0 | 0 | 3 | 1 |
| F | Nucleotide transport and metabolism | 1 | 0 | **8** | 2 | 0 | 0 | 0 | 1 |
| G | Carbohydrate transport and metabolism | 1 | 1 | 5 | **8** | 0 | (1) | 0 | 0 |
| H | Coenzyme transport and metabolism | **6** | 0 | 6 | 2 | 0 | 0 | 0 | 0 |
| I | Lipid transport and metabolism | 1 | 1 | 4 | **16** | 0 | 0 | 3 | 5 |
| P | Inorganic ion transport and metabolism | **9** | (2) | 4 | **8** | 0 | 0 | **11** | 1 |
| Q | Secondary metabolites biosynthesis; transport and catabolism | 0 | 0 | (3) | 1 | 0 | 0 | (1) | 0 |
|  | POORLY CHARACTERIZED | 30 | 13 | 82 | 34 | **10** | (1) | 18 | 11 |
| R | General function prediction only | **14** | 5 | 27 | 18 | 2 | 0 | 7 | 5 |
| S | Function unknown | 13 | **6** | 33 | 12 | 2 | (1) | 8 | 3 |
| X | Not predicted | 3 | 2 | 22 | 4 | **6** | 0 | 3 | 3 |

**Table S1** Distribution and significant enrichment of up-regulated genes and COG categories. Values that are above 4 and determined as significantly enriched by the Chi-squared test (p > 0.05) are highlighted in bold.

**Table S2** Selected significant differential expression (fold change for genes with p-value < 0.00001; ANOVA). The genes displayed are differentially regulated more than 1.5 fold and at-one-time expressed above average expression levels, (exceptions are included for genes of particular interest, in *italic*). Bar plots display absolute transcriptional abundance (0-3.6, scale shown header), variance corresponding to values of 2 standard deviance around the mean are displayed as darker color. Gray/black bars denote the total expression and variance in samples from Hocking et al. [13] (+), with results from Ø-CO, S-CO and T-CO denoted in successive bars. (Selected areas correspond to genes specifically addressed in the paper, colored according to in Figure 1 and 3, light gray denotes constitutive genes of general importance).

| Locus tag | Locus | NCBI annotation* | | | | | strand | | | | | | COG | | Fold change^⦁^ (ANOVA p< 0.00001) | | | | Expression^⦁^  +/Ø/S/T   |
| --- | --- | --- | --- | --- | --- | --- | --- | --- | --- | --- | --- | --- | --- | --- | --- | --- | --- | --- | --- |
|  |  |  |  |  |  |  |  |  |  |  |  |  |  |  | CO vs H.2014 | Ø-CO  vs S/T-CO | S-CO  vs Ø/T-CO | T-CO  vs Ø/S-CO |  |
| *AF0013* | *exuT* | hexuronate transporter | | | | | + | | | | | | G | |  | **-1.6** |  |  |  |
| AF0058 |  | Protein involved in ribosomal biogenesis, contains PuA domain* | | | | | - | | | | | | J | |  | **2.1** |  |  |  |
| AF0059 |  | uncharacterized conserved protein* | | | | | - | | | | | | S | |  | **2** |  |  |  |
| AF0071 |  | ATP-dependent RNA helicase, putative | | | | | - | | | | | | V | |  | **2** |  |  |  |
| AF0072 |  | CRISPR system related protein, RAMP superfamily* | | | | | - | | | | | | V | |  | **1.9** |  |  |  |
| AF0077 | *aor-2* | aldehyde ferredoxin oxidoreductase | | | | | + | | | | | | C | | **-1.6** |  |  |  |  |
| AF0108 |  | fructose-bisphosphate aldolase | | | | | - | | | | | | E | | **-1.6** |  |  |  |  |
| AF0117 | *act-1* | pyruvate formate-lyase activating enzyme | | | | | - | | | | | | O | |  | **1.7** |  |  |  |
| AF0118 |  | hypothetical protein | | | | | - | | | | | | X | |  | **1.6** |  |  |  |
| AF0126 |  | hypothetical protein | | | | | - | | | | | | X | |  |  | **13.8** |  |  |
| AF0127 |  | Predicted permease* | | | | | - | | | | | | R | |  |  |  | **2.7** |  |
| AF0141 |  | hypothetical protein | | | | | + | | | | | | X | |  |  | **14.1** |  |  |
| AF0142 |  | cytochrome C oxidase, subunit II, putative | | | | | + | | | | | | C | |  |  | **8.6** |  |  |
| AF0143 |  | Polyferredoxin* | | | | | + | | | | | | C | |  |  | **5** |  |  |
| *AF0144* | *cbaB* | cytochrome C oxidase, subunit II | | | | | + | | | | | | C | |  |  | **19.4** |  |  |
| *AF0145* |  | hypothetical protein | | | | | - | | | | | | X | |  |  | **6.5** |  |  |
| AF0154 |  | High-affinity Fe2+/Pb2+ permease* | | | | | + | | | | | | P | |  |  |  | **7.5** |  |
| AF0155 |  | hypothetical protein | | | | | + | | | | | | X | |  |  |  | **4** |  |
| *AF0156* | *fdx-1* | Ferredoxin | | | | | + | | | | | | C | |  |  |  | **2.4** |  |
| *AF0157* |  | molybdopterin oxidoreductase, iron-sulfur binding subunit | | | | | + | | | | | | C | |  |  |  | **2.2** |  |
| AF0158 |  | Predicted membrane protein* | | | | | + | | | | | | S | |  |  |  | 1.4 |  |
| AF0159 |  | molybdopterin oxidoreductase, molybdopterin binding subunit, putative | | | | | + | | | | | | C | |  |  |  |  |  |
| *AF0177* | *fwdE* | tungsten formylmethanofuran dehydrogenase, subunit E | | | | | - | | | | | | C | |  | 1.4 |  |  |  |
| AF0189 |  | hypothetical protein | | | | | - | | | | | | X | |  |  | **2** |  |  |
| *AF0190* |  | cytochrome C oxidase, subunit II, putative | | | | | - | | | | | | C | |  |  | **4.1** |  |  |
| AF0214 |  | S-layer domain* | | | | | + | | | | | | M | |  | **-1.9** |  |  |  |
| AF0218 | *trkA-1* | TRK potassium uptake system protein | | | | | - | | | | | | P | |  | **1.6** |  |  |  |
| *AF0219* | *leuA-2* | 2-isopropylmalate synthase | | | | | - | | | | | | E | |  | 1.5 |  |  |  |
| AF0221 | *braG-1* | branched-chain amino acid ABC transporter, ATP-binding protein | | | | | - | | | | | | E | |  |  |  | **-2.3** |  |
| AF0225 | *braE-1* | branched-chain amino acid ABC transporter, permease protein | | | | | + | | | | | | E | | **-1.7** |  |  |  |  |
| AF0226 | *noxC* | NADH oxidase | | | | | + | | | | | | C | |  |  | **3.3** |  |  |
| AF0227 | *pheA* | chorismate mutase/prephenate dehydratase | | | | | - | | | | | | E | |  | **1.6** |  |  |  |
| AF0228 | *aroD* | 3-dehydroquinate dehydratase | | | | | - | | | | | | E | |  | **1.8** |  |  |  |
| *AF0229* |  | 3-dehydroquinate synthase | | | | | - | | | | | | E | |  | 1.5 |  |  |  |
| *AF0230* |  | fructose-bisphosphate aldolase | | | | | - | | | | | | E | |  | 1.5 |  |  |  |
| AF0245 | *desR* | iron-dependent repressor | | | | | - | | | | | | K | |  | **-1.8** |  |  |  |
| AF0246 | *feoB-1* | iron transporter | | | | | - | | | | | | P | | **-2.2** | **-1.9** |  |  |  |
| *AF0251* |  | Ca2+/Na+ antiporter* | | | | | - | | | | | | P | | **1.73** | **-1.6** |  |  |  |
| AF0276 |  | NaMN:DMB phosphoribosyltransferase* | | | | | + | | | | | | H | |  | **-1.8** |  |  |  |
| AF0280 |  | Predicted RNA-binding protein, contains TRAM domain* | | | | | + | | | | | | R | |  | **1.8** |  |  |  |
| AF0333 | *hpaA-1* | 4-hydroxyphenylacetate-3-hydroxylase | | | | | + | | | | | | Q | |  | **1.9** |  |  |  |
| AF0343 | *wrbA* | tryptophan repressor binding protein | | | | | - | | | | | | R | | **-1.9** |  |  |  |  |
| AF0344 |  | desulfoferrodoxin, putative | | | | | - | | | | | | C | | **-1.8** |  |  |  |  |
| AF0355 | *fdx-3* | Ferredoxin | | | | | + | | | | | | C | |  | **-1.6** |  |  |  |
| AF0371 |  | N-glycosylase/DNA lyase | | | | | - | | | | | | L | | **1.7** |  |  |  |  |
| AF0372 |  | uncharacterized protein required for formate dehydrogenase activity* | | | | | - | | | | | | C | | **1.8** |  |  |  |  |
| *AF0376* | cdhE | acetyl-CoA decarbonylase/synthase complex subunit gamma | | | | | - | | | | | | C | |  |  |  |  |  |
| *AF0377* | cdhD | acetyl-CoA decarbonylase/synthase complex subunit delta | | | | | - | | | | | | C | |  |  |  |  |  |
| *AF0379* | cdhC | acetyl-CoA decarbonylase/synthase complex subunit beta | | | | | - | | | | | | C | |  |  |  |  |  |
| AF0392 |  | Predicted permease* | | | | | - | | | | | | R | |  |  |  | **-1.8** |  |
| *AF0394* | *Dld* | D-lactate dehydrogenase, cytochrome-type | | | | | - | | | | | | C | |  | **-1.6** |  |  |  |
| *AF0395* | *noxA-2* | NADH oxidase | | | | | - | | | | | | P | |  |  |  |  |  |
| *AF0401* |  | carbohydrate kinase | | | | | + | | | | | | G | |  | **-2** |  |  |  |
| *AF0407* |  | uncharacterized conserved protein* | | | | | - | | | | | | S | |  | 1.5 |  |  |  |
| AF0408 |  | uncharacterized conserved protein* | | | | | - | | | | | | S | | **1.6** | 1.5 |  |  |  |
| AF0420 |  | uncharacterized conserved protein* | | | | | - | | | | | | S | | **1.9** | 1.4 |  |  |  |
| *AF0423* | *dsrA* | sulfite reductase, subunit alpha | | | | | + | | | | | | C | |  |  |  |  |  |
| *AF0424* | *dsrB* | sulfite reductase, subunit beta | | | | | + | | | | | | C | |  |  |  |  |  |
| *AF0425* | *dsrD* | sulfite reductase, subunit gamma | | | | | + | | | | | | C | |  |  |  |  |  |
| *AF0429* |  | Methyltransferase | | | | | - | | | | | | Q | |  |  |  | 1.5 |  |
| AF0430 | *hemV-1* | iron ABC transporter, ATP-binding protein | | | | | - | | | | | | P | |  |  |  | **1.7** |  |
| AF0431 | *hemU-1* | iron ABC transporter, permease protein | | | | | - | | | | | | P | |  |  |  | **1.7** |  |
| AF0432 | *hemV-2* | iron ABC transporter, ATP-binding protein | | | | | - | | | | | | P | |  |  |  | **1.6** |  |
| AF0433 |  | uncharacterized conserved protein* | | | | | - | | | | | | S | |  |  |  | **2** |  |
| *AF0458* | *Pmm* | Phosphomannomutase | | | | | - | | | | | | G | |  | -1.5 |  |  |  |
| AF0473 | *pacS* | cation-transporting ATPase, P-type | | | | | - | | | | | | P | |  |  |  | **-1.8** |  |
| *AF0499* | *dsrO^i^* | molybdopterin oxidoreductase, iron-sulfur binding subunit | | | | | + | | | | | | C | |  |  |  |  |  |
| *AF0500* | *dsrP^i^* | molybdopterin oxidoreductase, membrane subunit | | | | | + | | | | | | C | |  |  |  |  |  |
| *AF0501* | *dsrM^i^* | nitrate reductase, gamma subunit, putative | | | | | + | | | | | | C | |  |  |  |  |  |
| *AF0502* | *dsrK^i^* | heterodisulfide reductase, subunit D, putative | | | | | + | | | | | | C | |  |  |  |  |  |
| *AF0503* | *dsrJ^i^* | uncharacterized conserved protein* | | | | | + | | | | | | S | |  |  |  |  |  |
| AF0533 |  | Icc family phosphoesterase* | | | | | + | | | | | | R | |  | **1.6** |  |  |  |
| *AF0543* | *dsrK^i^* | Fe-S oxidoreductase* | | | | | - | | | | | | C | |  |  |  |  |  |
| *AF0544* | *dsrK^i^* | Fe-S oxidoreductase* | | | | | - | | | | | | C | |  |  |  |  |  |
| *AF0545* | *dsrM^i^* | Nitrate reductase gamma subunit* | | | | | - | | | | | | C | |  |  |  |  |  |
| AF0546 | *dsrM^i^* | nitrate reductase, gamma subunit | | | | | + | | | | | | C | |  | **4.3** |  |  |  |
| AF0547 | *dsrK^i^* | reductase, iron-sulfur binding subunit | | | | | + | | | | | | C | |  | **4.1** |  |  |  |
| AF0576 |  | hypothetical protein | | | | | + | | | | | | X | | **1.6** |  |  |  |  |
| AF0577 |  | Radical SAM superfamily enzyme* | | | | | + | | | | | | R | | **2.3** |  |  |  |  |
| AF0590 | *hisG* | ATP phosphoribosyltransferase | | | | | + | | | | | | E | | **1.57** |  |  |  |  |
| AF0624 |  | uncharacterized membrane protein* | | | | | + | | | | | | S | |  | **-1.7** |  |  |  |
| *AF0661* | qmoC^i^ | heterodisulfide reductase, subunit E, putative | | | | | - | | | | | | C | |  |  |  |  |  |
| *AF0662* | qmoB^i^ | heterodisulfide reductase, subunit A | | | | | - | | | | | | C | |  |  |  |  |  |
| *AF0663* | qmoA^i^ | heterodisulfide reductase, subunit A | | | | | - | | | | | | C | |  | -1.1 |  |  |  |
| *AF0714* | *Mtd* | F420-dependent methylenetetrahydromethanopterin dehydrogenase | | | | | + | | | | | | C | |  | -1.3 |  |  |  |
| *AF0734* |  | ribosome biogenesis protein | | | | | - | | | | | | J | | **1.85** | 1.5 |  |  |  |
| AF0736 |  | Predicted membrane protein* | | | | | + | | | | | | S | | **2.2** |  |  |  |  |
| AF0747 | *dapF* | diaminopimelate epimerase | | | | | - | | | | | | E | | **1.51** |  |  |  |  |
| AF0755 | *hdrDE^i^* | heterodisulfide reductase, subunits E and D, putative | | | | | - | | | | | | C | |  |  |  | **3.6** |  |
| AF0787 |  | Predicted cation transporter* | | | | | + | | | | | | R | |  | **-2.2** |  |  |  |
| AF0788 |  | Permease of the drug/metabolite transporter (DMT) superfamily* | | | | | + | | | | | | G | |  | **-1.8** |  |  |  |
| AF0793 |  | uncharacterized conserved protein* | | | | | + | | | | | | S | |  | **1.7** |  |  |  |
| *AF0806* | *lctP* | L-lactate permease | | | | | + | | | | | | C | |  |  |  |  |  |
| *AF0807* | *lldD* | L-lactate dehydrogenase, cytochrome-type | | | | | - | | | | | | H | |  |  |  |  |  |
| AF0808 | *dld^i^* | glycolate oxidase subunit | | | | | + | | | | | | C | |  | **1.8** |  |  |  |
| *AF0809* | *lldE^i^* | heterodisulfide reductase, subunit D, putative | | | | | + | | | | | | C | |  |  |  |  |  |
| AF0810 | *lldG^i^* | hypothetical protein | | | | | + | | | | | | X | |  | **1.8** |  |  |  |
| AF0811 | *lldF^i^* | uncharacterized conserved protein containing ferredoxin-like domain* | | | | | + | | | | | | C | |  | **1.9** |  |  |  |
| AF0827 | *braC-2* | branched-chain amino acid ABC transporter, periplasmic binding protein | | | | | - | | | | | | E | |  | **1.8** |  |  |  |
| *AF0905* |  | Ribonuclease M5 (contains TOPRIM domain)* | | | | | - | | | | | | L | |  | **1.7** |  |  |  |
| AF0950 | *cooF* | carbon monoxide dehydrogenase, iron sulfur subunit | | | | | + | | | | | | C | | **1.6** | 1.2 |  |  |  |
| *AF0951* | *noxA-4* | NADH oxidase | | | | | + | | | | | | R | | 1.4 |  |  |  |  |
| AF0963 | *fad-3* | enoyl-CoA hydratase | | | | | - | | | | | | I | |  | **-2.6** |  |  |  |
| AF0964 | *acd-6* | acyl-CoA dehydrogenase | | | | | - | | | | | | I | |  | **-2.1** |  |  |  |
| AF0967 | *acaB-9* | acetyl-CoA acetyltransferase | | | | | - | | | | | | I | |  | **-2** |  |  |  |
| AF0977 | *amt-1* | ammonium transporter | | | | | + | | | | | | P | | **2.4** |  |  |  |  |
| AF0990 | *caiB-2* | L-carnitine dehydratase | | | | | - | | | | | | C | |  |  |  | **1.6** |  |
| AF0991 | *gcdH* | glutaryl-CoA dehydrogenase0 | | | | | - | | | | | | I | | **-3.1** |  |  | **1.8** |  |
| AF1025 | *hbd-4* | 3-hydroxyacyl-CoA dehydrogenase | | | | | - | | | | | | I | |  | **1.6** |  |  |  |
| AF1029 | *fadD-5* | long-chain-fatty-acid--CoA ligase | | | | | - | | | | | | I | |  | **1.6** |  |  |  |
| AF1052 |  | Putative archaeal flagellar protein G* | | | | | - | | | | | | N | |  | -1.5 |  |  |  |
| AF1066 | *mer-1* | methylenetetrahydromethanopterin reductase | | | | | + | | | | | | C | |  | **-1.6** |  |  |  |
| AF1098 | *fum-1* | fumarate hydratase | | | | | - | | | | | | C | |  | **-1.9** |  |  |  |
| AF1099 | *fum-2* | fumarate hydratase | | | | | - | | | | | | C | |  | **-2.3** |  |  |  |
| AF1100 | *cdhA-1* | acetyl-CoA decarbonylase/synthase complex subunit alpha | | | | | + | | | | | | C | |  |  |  | **2.5** |  |
| *AF1101* | *cdhB-1* | acetyl-CoA decarbonylase/synthase complex subunit epsilon | | | | | + | | | | | | C | |  |  |  |  |  |
| AF1102 |  | uncharacterized conserved protein* | | | | | + | | | | | | S | |  |  |  | **2.3** |  |
| *AF1140* |  | Predicted permease* | | | | | + | | | | | | R | |  | 1.5 |  |  |  |
| *AF1194* |  | Predicted HTH domain, homologous to N-terminal domain of RPA1 protein family* | | | | | - | | | | | | R | |  | 1.5 |  |  |  |
| *AF1196* | *mer-2* | N5,N10-methylenetetrahydromethanopterin reductase | | | | | - | | | | | | C | |  |  |  |  |  |
| AF1236 |  | hypothetical protein | | | | | + | | | | | | X | |  | **15.1** |  |  |  |
| AF1237 |  | Membrane-bound tetraheme cytochrome c subunit* | | | | | - | | | | | | C | |  | **18.8** |  |  |  |
| AF1247 |  | 2-methylthioadenine synthetase* | | | | | + | | | | | | J | | **1.81** | 1.4 |  |  |  |
| AF1252m | *oadA* | oxaloacetate decarboxylase | | | | | - | | | | | | C | |  | **-1.6** |  |  |  |
| AF1254 |  | Acetyltransferase (GNAT) family* | | | | | + | | | | | | K | |  | **1.6** |  |  |  |
| AF1285 |  | cell division protein CDC48 | | | | | - | | | | | | R | |  | **1.8** |  |  |  |
| AF1287 | *acs-6* | acetyl-CoA synthetase | | | | | - | | | | | | I | |  | **-2.2** |  |  |  |
| AF1288a |  | methylmalonyl-CoA mutase N-terminal domain-containing protein | | | | | + | | | | | | I | |  | **-1.7** |  |  |  |
| AF1288b |  | methylmalonyl-CoA mutase C-terminal domain-containing protein | | | | | + | | | | | | I | |  | -1.5 |  |  |  |
| AF1315 | *baiF-3* | bile acid-inducible operon protein F | | | | | - | | | | | | C | |  | **-1.6** |  |  |  |
| AF1322 |  | Predicted membrane-associated Zn-dependent protease* | | | | | - | | | | | | M | |  | -1.5 |  |  |  |
| AF1356 | *phoX* | phosphate ABC transporter, periplasmic phosphate-binding protein | | | | | + | | | | | | P | | **3.6** | **-1.9** |  |  |  |
| AF1357 | *pstC* | phosphate ABC transporter, permease protein | | | | | + | | | | | | P | | **3.9** | **-1.9** |  |  |  |
| AF1358 | *pstA* | phosphate ABC transporter, permease protein | | | | | + | | | | | | P | | **3.1** | **-1.6** |  |  |  |
| AF1359 | *pstB* | phosphate ABC transporter, ATP-binding protein | | | | | + | | | | | | P | | **3.3** | **-1.6** |  |  |  |
| AF1360 | *phoU* | | | phosphate ABC transporter, regulatory protein | | | + | | | | | | P | | **2.2** |  |  |  |  |
| AF1361 | *arsC* | arsenate reductase | | | | | + | | | | | | T | | **1.9** |  |  |  |  |
| AF1392 | *braD-4* | branched-chain amino acid ABC transporter, permease protein | | | | | + | | | | | | E | |  | **-1.6** |  |  |  |
| AF1393 | *braE-4* | branched-chain amino acid ABC transporter, permease protein | | | | | + | | | | | | E | |  | -1.5 |  |  |  |
| AF1464 |  | uncharacterized conserved protein* | | | | | + | | | | | | S | |  |  | **-1.8** |  |  |
| AF1504 |  | hypothetical protein | | | | | - | | | | | | X | |  | **-1.7** |  |  |  |
| *AF1534* |  | Putative sterol carrier protein* | | | | | + | | | | | | I | |  |  |  | **1.7** |  |
| AF1535 | *ftrB* | ferredoxin-thioredoxin reductase, catalytic subunit | | | | | - | | | | | | C | |  |  |  | **2.7** |  |
| AF1536 | *grx-1* | Glutaredoxin | | | | | - | | | | | | O | |  |  |  | **2.3** |  |
| AF1573 |  | uncharacterized conserved protein* | | | | | + | | | | | | S | |  | **1.9** |  |  |  |
| *AF1574* |  | uncharacterized conserved protein* | | | | | + | | | | | | S | |  | 1.3 |  |  |  |
| AF1575 |  | uncharacterized conserved protein* | | | | | + | | | | | | S | |  | **2** |  |  |  |
| AF1576 |  | hypothetical protein | | | | | + | | | | | | X | |  | **2.2** |  |  |  |
| *AF1597* |  | hypothetical protein | | | | | - | | | | | | X | |  |  |  | **-1.9** |  |
| *AF1598* |  | uncharacterized conserved protein* | | | | | - | | | | | | S | |  |  |  | **-1.6** |  |
| AF1616 |  | uncharacterized conserved protein* | | | | | - | | | | | | S | |  | **1.9** |  |  |  |
| *AF1649* | *fwdG* | tungsten formylmethanofuran dehydrogenase, subunit G | | | | | + | | | | | | C | |  |  |  |  |  |
| AF1650 | *fwdB-1* | tungsten formylmethanofuran dehydrogenase, subunit B | | | | | + | | | | | | C | |  | **-2.5** |  |  |  |
| *AF1651* | *fwdD-1* | tungsten formylmethanofuran dehydrogenase, subunit D | | | | | + | | | | | | C | |  |  |  |  |  |
| *AF1667* | Sat | sulfate adenylyltransferase | | | | | + | | | | | | P | |  |  |  |  |  |
| *AF1668* |  | uncharacterized conserved protein* | | | | | + | | | | | | S | |  |  |  |  |  |
| *AF1669* | aprB | adenylylsulfate reductase, subunit B | | | | | + | | | | | | C | |  |  |  |  |  |
| *AF1670* | aprA | adenylylsulfate reductase | | | | | + | | | | | | C | |  |  |  | -1.1 |  |
| *AF1736* | *mvaA* | 3-hydroxy-3-methylglutaryl-coenzyme A reductase | | | | | - | | | | | | I | |  | -1.5 |  |  |  |
| AF1760 |  | Nucleotide-binding protein, uspA family* | | | | | + | | | | | | T | |  | **-1.6** |  |  |  |
| AF1823 |  | F420H2:quinone oxidoreductase, 16.5 kDa subunit, putative | | | | | + | | | | | | S | |  | **-2.7** |  |  |  |
| *AF1824* |  | F420H2:quinone oxidoreductase, 11.2 kDa subunit, putative | | | | | + | | | | | | C | |  |  |  |  |  |
| *AF1825* | *nuoM* | | | F420H2:quinone oxidoreductase, 53.9 kDa subunit | | | + | | | | | | C | |  |  |  |  |  |
| *AF1826* | *nuoL* | F420H2:quinone oxidoreductase, 72.4 kDa subunit. | | | | | + | | | | | | C | |  | -1.4 |  |  |  |
| *AF1827* |  | F420H2:quinone oxidoreductase, 43.2 kDa subunit, putative | | | | | + | | | | | | C | |  | -1.1 |  |  |  |
| *AF1828* |  | NADH dehydrogenase subunit A | | | | | + | | | | | | C | |  | -1.1 |  |  |  |
| *AF1829* |  | F420H2:quinone oxidoreductase, 39.7 kDa subunit, putative | | | | | + | | | | | | C | |  |  |  |  |  |
| *AF1830* | *nuoD* | NADH dehydrogenase subunit D | | | | | + | | | | | | C | |  | -1.1 |  |  |  |
| *AF1831* |  | NADH dehydrogenase subunit H | | | | | + | | | | | | C | |  | -1.1 |  |  |  |
| *AF1832a* |  | NADH dehydrogenase subunit I | | | | | + | | | | | | C | |  |  |  |  |  |
| *AF1833* |  | F420H2:quinone oxidoreductase, 39 kDa subunit, putative | | | | | + | | | | | | C | |  |  |  |  |  |
| AF1843 | *cbiM-2* | | | cobalamin biosynthesis protein | | | - | | | | | | P | |  |  |  | **2** |  |
| *AF1849* | *cooS* | carbon monoxide dehydrogenase, catalytic subunit | | | | | + | | | | | | C | |  |  |  |  |  |
| AF1856 |  | uncharacterized conserved protein* | | | | | + | | | | | | S | |  |  |  | **2.8** |  |
| *AF1877* |  | CRISPR-associated protein Cas4, RecB family exonuclease* | | | | | - | | | | | | V | |  | **1.8** |  |  |  |
| *AF1878* |  | CRISPR-associated protein Cas1* | | | | | - | | | | | | V | | **-2.77** | **3.5** |  |  |  |
| *AF1879* |  | CRISPR-associated protein, RecB family exonuclease* | | | | | - | | | | | | V | | **-2.75** | **3.6** |  |  |  |
| AF1897 |  | Glycosyltransferase* | | | | | - | | | | | | M | |  | **1.6** |  |  |  |
| AF1901 |  | OxaA/SpoJ/YigC translocase/secretase, sec-independent itegration of nascent memrane proteins into membrane* | | | | | - | | | | | | U | |  | **-1.9** |  |  |  |
| *AF1928* | *fwdD-2* | tungsten formylmethanofuran dehydrogenase, subunit D | | | | | + | | | | | | C | |  | -1.2 |  |  |  |
| *AF1929* | *fwdB-2* | tungsten formylmethanofuran dehydrogenase, subunit B | | | | | + | | | | | | C | |  |  |  |  |  |
| *AF1930* | *fwdA* | tungsten formylmethanofuran dehydrogenase, subunit A | | | | | + | | | | | | C | |  |  |  |  |  |
| *AF1931* | *fwdC* | tungsten formylmethanofuran dehydrogenase, subunit C | | | | | + | | | | | | C | |  |  |  |  |  |
| *AF1935* | *Mch* | N(5),N(10)-methenyltetrahydromethanopterin cyclohydrolase | | | | | - | | | | | | H | |  |  |  |  |  |
| AF1981 |  | ABC transporter, permease protein | | | | | - | | | | | | P | |  |  |  | **1.9** |  |
| AF1982 |  | ABC transporter, ATP-binding protein | | | | | - | | | | | | P | |  |  |  | **1.8** |  |
| AF1983 |  | ABC transporter, periplasmic binding protein | | | | | - | | | | | | P | |  |  |  | **1.7** |  |
| AF1984 | *troR* | iron-dependent repressor | | | | | - | | | | | | K | |  |  |  | **2** |  |
| *AF2073* | ftr-1 | tetrahydromethanopterin formyltransferase | | | | | - | | | | | | C | |  |  |  |  |  |
| AF2159 |  | uncharacterized conserved protein* | | | | | - | | | | | | S | | **-1.9** |  |  |  |  |
| AF2167 |  | hypothetical protein | | | | | - | | | | | | X | | **-1.6** |  |  |  |  |
| AF2170 |  | uncharacterized conserved protein* | | | | | - | | | | | | S | |  | **1.7** |  |  |  |
| *AF2207* | *ftr-*2 | tetrahydromethanopterin formyltransferase | | | | | | + | | | | C | | |  |  |  |  |  |
| AF2219 | *mcmA2* | methylmalonyl-CoA mutase, subunit alpha, C-terminus | | | - | | | | I | | | | |  | | **-1.8** |  |  |  |
| AF2228 | dsvC | sulfite reductase, desulfoviridin-type subunit gamma | | | | + | | | | | P | | |  | |  |  |  |  |
| AF2237 |  | HAM1 protein | | | | | | | | + | | F | |  | | 1.5 |  |  |  |
| AF2243 | *fadA-3* | acetyl-CoA acetyltransferase | | | | | | | | - | | I | |  | | **-1.8** |  |  |  |
| AF2244 | *acd-11* | acyl-CoA dehydrogenase | | | | | | | | - | | I | |  | | **-2** |  |  |  |
| AF2302 |  | uncharacterized alpha+beta fold domain often fused to a RimK-like ATP-grasp enzyme* | | | | | | | | + | | S | | **2.1** | |  |  |  |  |
| AF2380 |  | iron-sulfur cluster binding protein | | | | | | | | + | | C | |  | | **-1.6** |  |  |  |
| AF2381 |  | iron-sulfur cluster binding protein | | | | | | | | + | | C | |  | |  |  | **1.8** |  |
| AF2382 |  | nucleotide-binding protein | | | | | | | | + | | D | |  | |  |  | **2.1** |  |
| *AF2383* |  | Metal-dependent hydrolase of the beta-lactamase superfamily II* | | | | | | | | + | | R | |  | |  |  | **1.7** |  |
| AF2384 |  | molybdopterin oxidoreductase, molybdopterin binding subunit | | | | | | | | + | | C | |  | |  |  | **1.8** |  |
| AF2385 |  | molybdopterin oxidoreductase, iron-sulfur binding subunit | | | | | | | | + | | C | |  | |  |  | **3.4** |  |
| AF2386 |  | molybdopterin oxidoreductase, membrane subunit | | | | | | | | + | | P | |  | |  |  | **3.1** |  |
| AF2392 |  | uncharacterized conserved protein* | | | | | | | | + | | S | |  | |  |  | **2.3** |  |
| AF2394 | *feoB-2* | | iron (II) transporter | | | | | | | - | | P | |  | |  |  | **1.6** |  |
| AF2414 |  | Predicted transcriptional regulator* | | | | | | | | + | | K | |  | |  |  | **-1.6** |  |
| *AF2397* | *cdhA-2* | acetyl-CoA decarbonylase/synthase complex subunit alpha | | | | | | | | + | | C | |  | |  |  |  |  |
| *AF2398* | *cdhB-2* | acetyl-CoA decarbonylase/synthase complex subunit epsilon | | | | | | | | + | | C | |  | |  |  |  |  |
| *AF2415* | *acaA-2* | 3-hydroxy-3-methylglutaryl CoA synthase* | | | | | | | | + | | I | |  | |  |  | -1.4 |  |
| *AF2416* | *acaB-12* | acetyl-CoA acetyltransferase | | | | | | | | + | | I | |  | |  |  | -1.5 |  |
| *AF2417* |  | Predicted nucleic-acid-binding protein containing a Zn-ribbon* | | | | | | | | + | | R | |  | |  |  | **-1.8** |  |

^⦁^ For full data: Array Express, accession number E-MTAB-3035; H.2014, E-MTAB-2294 (www.ebi.ac.uk/arrayexpress)

*arCOG annotation; Wolf et al., 2012

^i^ infered locus annotation

**H.2014 -** data from Hocking et al. 2014

**Table S3** Overview of transcription profiles of acetyl-CoA synthetase genes, se Table S2 for descriptions.

| Locus tag | Locus | NCBI annotation* | strand | COG | Fold change (ANOVA p< 0.00001) | | | | | | | Expression  +/Ø/S/T |
| --- | --- | --- | --- | --- | --- | --- | --- | --- | --- | --- | --- | --- |
|  |  |  |  |  | CO vs  H.2014 | Ø-CO vs S/T-CO | | S-CO vs Ø/T-CO | | T-CO  vs Ø/S-CO | |  |
| Acs: *PP_i_ + AMP + Acetyl-CoA ⇌ ATP + CoA + Acetate* | | | | | | | | | | | | |
| *AF0197* | acs-1 | acetoacetyl-CoA synthetase | + | I |  |  |  | |  | |  | |
| *AF0366* | acs-2 | acetyl-CoA synthetase | + | I |  |  |  | |  | |  | |
| AF0677 | acs-3 | acetyl-CoA synthetase | - | I |  |  |  | |  | |  | |
| AF0975 | acs-4 | acetyl-CoA synthetase | + | I |  |  |  | |  | |  | |
| AF0976 | acs-5 | acetyl-CoA synthetase | + | I |  |  |  | |  | |  | |
| AF1287 | acs-6 | acetyl-CoA synthetase | - | I |  | **-2.2** |  | |  | |  | |
| *AF2389-C* | AF2389-N | acetyl-CoA synthetase, putative | + | I |  |  |  | |  | |  | |

**Table S4:** Primers and probes used for TaqMan® quantitative real-time PCR analyses

| Locus tag | Locus | Primers;  (forward, reverse, probe) |
| --- | --- | --- |
| AF0424 | *dsrB* | **FW:**ACGACGAGGCCATCAGAAAG **RV:**TTGGACATGCAGCCACTGTT **PB:**CCTGTGAGATTCCG |
| (AF1101) | *cdhB-1* | **FW:**TGGTGGAGTTTGCAGTGAAGTT **RV:**GCCTGCCGTTGCTGCTA **PB:**CTGAAAAAGGGATTCCG |
| (AF2398) | *cdhB-2* | **FW:**AGTGCCATCACGAGGTTCATT **RV:**AAGGACCGCGTAGTTCACCTT **PB:**ATGCTGGTTTGGGCG |
| (AF0377) | *cdhD* | **FW:**GGCCCATGCTCGCAAAG **RV:**AGGGTTGTCCAGCACATCCT **PB:**CGGTGAGGATGCACTAC |
| (AF1849) | *cooS* | **FW:**TGCATACGGCCTCACAACTC **RV:**ATCCTCGCTTCCCGTGATT **PB:**CGTTTCGCCTGTTCC |
| (AF1670) | *aprA* | **FW:**TGACTACGCAAGGCATGTTGA **RV:**TGGGCAGTCCCCACTTCTC **PB:**ACGGTCCACCTCTT |
| AF1667 | *Sat* | **FW:**TACTCTGGAGCCAGGGCATT **RV:**CCTCATTGGGCGGAGCTT **PB:**CGAGCTTCGGCTTC |
